# Indian Traditional Ayurvedic System of Medicine and Nutritional Supplementation

**DOI:** 10.1155/2013/376327

**Published:** 2013-06-23

**Authors:** M. M. Pandey, Subha Rastogi, A. K. S. Rawat

**Affiliations:** Pharmacognosy & Ethnopharmacology Division, CSIR-National Botanical Research Institute, Rana Pratap Marg, Lucknow 226001, India

## Abstract

Food is the major source for serving the nutritional needs, but with growing modernization some traditional ways are being given up. Affluence of working population with changing lifestyles and reducing affordability of sick care, in terms of time and money involved, are some of the forces that are presently driving people towards thinking about their wellness. There has been increased global interest in traditional medicine. Efforts to monitor and regulate traditional herbal medicine are underway. Ayurveda, the traditional Indian medicine, remains the most ancient yet living traditions. Although India has been successful in promoting its therapies with more research and science-based approach, it still needs more extensive research and evidence base. Increased side effects, lack of curative treatment for several chronic diseases, high cost of new drugs, microbial resistance and emerging, diseases are some reasons for renewed public interest in complementary and alternative medicines. Numerous nutraceutical combinations have entered the international market through exploration of ethnopharmacological claims made by different traditional practices. This review gives an overview of the Ayurvedic system of medicine and its role in translational medicine in order to overcome malnutrition and related disorders.

## 1. Introduction

India is known for its traditional medicinal systems—Ayurveda, Siddha, and Unani. Medical systems are found mentioned even in the ancient Vedas and other scriptures. The Ayurvedic concept appeared and developed between 2500 and 500 BC in India [1]. The literal meaning of Ayurveda is “science of life,” because ancient Indian system of health care focused on views of man and his illness. It has been pointed out that the positive health means metabolically well-balanced human beings. Ayurveda is also called the “science of longevity” because it offers a complete system to live a long healthy life. It offers programs to rejuvenate the body through diet and nutrition. It offers treatment methods to cure many common diseases such as food allergies, which have few modern treatments. However, one should be aware that Ayurvedic nutrition is not a “magic bullet” system but requires the full participation of the patient to succeed. It is an interactive system that is user-friendly and educational. It teaches the patient to become responsible and self-empowered. Ayurveda is not a nutritional system for those seeking an escape or excuse to further abuse their body or mind. It is a system for empowerment, a system of freedom, and long life.

Food is the major source for serving the nutritional needs, but with growing modernization some traditional methods are being given up (Table 1). Hence, the modern food habits are affecting the balanced nutrition [2]. There is an ever widening gap in nutrient intake due to which normal life is no longer normal. However, affluence of working population with changing lifestyles and reducing affordability of sick care, in terms of time and money involved, are some of the forces that are presently driving people towards thinking about their wellness.

## 2. Medicinal Plants Used in Alternative/Traditional Medicines

Alternative medicines are being used by about 60 percent of the world's population. These medicines are not only used by the rural masses for their primary health care in developing countries but are also used in developed countries where modern medicines dominate [3]. The Indian subcontinent is a vast repository of medicinal plants that are used in traditional medical treatments. The alternative medicines in the traditional systems are derived from herbs, minerals, and organic matter, while for the preparation of herbal drugs only medicinal plants are used. Use of plants as a source of medicine has been an ancient practice and is an important component of the health care system in India. In India, about 70 percent of rural population depends on the traditional Ayurvedic system of medicine. Most healers/practitioners of the traditional systems of medicine prepare formulations by their own recipes and dispense to the patients. In the Western countries, approximately 40 per cent of people are using the herbal medicine for the treatment of various diseases. This interest in traditional medicines is growing rapidly due to the attention being given to it by the governmental agencies and different NGO's comprising of general public and researchers as well as the increased side effects, adverse drug reactions, and cost factor of the modern medicines. 

India is the largest producer of medicinal plants. There are currently about 250,000 registered medical practitioners of the Ayurvedic system, as compared to about 700,000 of the modern medicine. In India, around 20,000 medicinal plants have been recorded; however, traditional practitioners use only 7,000–7,500 plants for curing different diseases. The proportion of use of plants in the different Indian systems of medicine is Ayurveda 2000, Siddha 1300, Unani 1000, Homeopathy 800, Tibetan 500, Modern 200, and folk 4500. In India, around 25,000 effective plant-based formulations are used in traditional and folk medicine. More than 1.5 million practitioners are using the traditional medicinal system for health care in India. It is estimated that more than 7800 manufacturing units are involved in the production of natural health products and traditional plant-based formulations in India, which requires more than 2000 tons of medicinal plant raw material annually [4]. More than 1500 herbals are sold as dietary supplements or ethnic traditional medicines [5].

Alternative medicines are being used by those people who do not use or cannot be helped by conventional medicinal system. Some common medicinal plants having nutraceutical potential and their primary use in traditional medicine [6–26] are being given in Table 2. 

## 3. Expanding Complementary and Alternative (CAM) Approaches

More than 80 percent of people in developing countries cannot afford the most basic medical procedures, drugs, and vaccines. Among wealthier populations in both developed and developing countries, complementary and alternative practices are popular although proof of their safety and effectiveness is modest. Evidence-based research in Ayurveda is receiving larger acceptance in India and abroad [27–30]. The National Center for Complementary and Alternative Medicine has been inaugurated as the United States Federal Government's lead agency for scientific research in this arena of medicine. Its mission is to explore complementary and alternative healing practices in the context of rigorous science, support sophisticated research, train researchers, disseminate information to the public on the modalities that work, and explain the scientific rationale underlying discoveries. The center is committed to explore and fund all such therapies for which there is sufficient preliminary data, compelling public health need and ethical justifications [31, 32].

Complementary and alternative practices are adjuncts or alternatives to Western medical approaches. Economic factors influence user behavior. Although social, cultural, and medical reasons account for most of the appeal of traditional approaches, economic factors also play a role. It is assumed that users of these approaches choose them because they are cheaper than conventional therapies or systems. However, several studies have found that CAM approaches cost the same or more than conventional treatments for the same conditions; thus, people seek them out for reasons other than cost. At least one study showed that financial factors ranked behind such reasons as confidence in the treatment, ease of access, and convenience, in the choice of a traditional healer. Another common misconception is that the poor are more likely to use traditional medicine, but this is not always true. Nowadays people seek CAM techniques because they believe the side effects will be lower. In both developed and developing countries, users of complementary methods also commonly seek conventional care [33].  Table 3 enlists some important Ayurvedic herbal formulations [34].

## 4. Nutraceuticals an Evolving Alternative Approach

**Figure 1:**
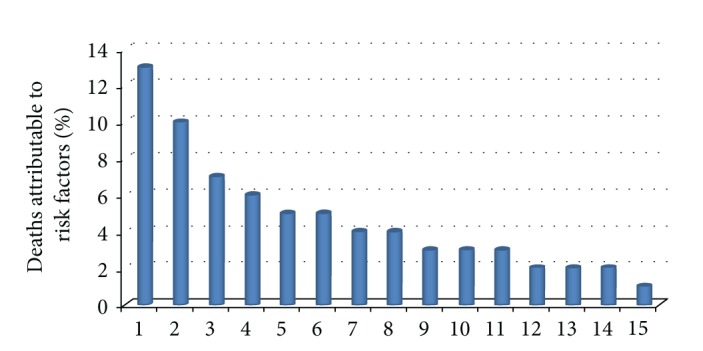
Risk factors related to nutrition— 1: underweight, 2: unsafe sex, 3: blood pressure, 4: unsafe water, sanitation and hygiene, 5: cholesterol, 6: tobacco, 7: indoor smoke from solid fuels, 8: low fruit and vegetable intake, 9: zinc deficiency, 10: iron deficiency, 11: vitamin A deficiency, 12: physical inactivity, 13: alcohol, 14: overweight, and 15: unsafe healthcare injections.

Nutrition is a fundamental need. Various risk factors related to health result from an imbalance in nutrition. These imbalances in India are widely prevalent leading to adverse outcomes. A certain section of the population consumes diet which does not provide sufficient calories, let alone sufficient nutrients. In India, nearly 20% of the total population and 44% of young children (below 5 years of age) are undernourished and underweight. On the other hand, there is a huge population that is nourished in calorie intake but not in terms of nutrient intake. This segment would typically include lower middle to upper class population with sufficient purchasing capacity but probably less awareness about their nutrient requirements, leading to imbalanced nutritional uptake. In fact, in our population about 30% in urban and 34% in rural areas consume more than the recommended number of calories with higher than recommended levels of dietary fats and could be the largest contributor in making India the future cardiovascular and diabetes capital of the world. The third population segment, which is about 80 million, consumes nutrients and calories more than those recommended for the lifestyle they have opted for. The main risk factors in developing countries like India are related to nutrition and contribute to nearly 40% of total death and 39% of total disease burden. The main leading risk factors in developing countries [2] are shown in Figure 1. 

According to WHO report, India has the largest burden of cardiovascular diseases and largest number of diabetes patients in the world. The number of cardiovascular diseases patients in Brazil, Russia, China, and India are 4.1, 11.8, 24.5, and 28.9 million, respectively. Likewise the numbers of diabetes patients in same countries are 4.6, 4.6, 20.8, and 31.7 million, respectively. An estimate of the cost of productivity lost on account of mortality due to nutrition-related disorders was estimated to be 0.85% of the GDP in 2004 and is expected to increase up to 1.2% for India's GDP by 2015. Nearly 340 million people, 30% of the population in urban areas and 34% of the population in rural areas, consume calories more than the norms. Even in the population that shows sufficient calorie intake, the micronutrient consumption is not at desired levels. While the intake of calorie-rich foods may be high, micronutrient-rich foods are being consumed in low proportions. As a result, significant micronutrient deficiencies exist in urban as well as rural areas [35].

Hence, the requirement of external intervention, that can supplement diet to help prevent nutrition-related disorders and promote wellness over treatment of various diseases, has become a necessity, and such products are known as nutraceuticals. A nutraceutical is a food or food component that claims to have health benefits, including treatment and prevention of disease. Nutraceuticals, an emerging concept, can be broadly categorized as products which are extracted from natural sources (nature-like) or manufactured synthetically (man-made), which supplement the diet to provide nutrition over and above regular food and help prevent nutrition-related disorders. Nutraceuticals, foods or food components that help in prevention or treatment of disease, are made from herbal/botanical raw materials. They do more than just supplement the diet. They, as was pointed out, help with disease prevention and treatment. Theoretically, the appeal of nutraceuticals is to accomplish treatment goals without side effects.

The nutraceutical industry is rapidly growing (7%–12% per year). With extensive anecdotal data on exciting health results, nutraceuticals promise significant contributions to disease prevention. The global nutraceuticals market is estimated at 117 billion US dollar of which India's share is a meager 0.9%. United States and Japan are key markets for nutraceutical consumption. Indian nutraceuticals market is about 1 billion USD which is increasing day by day. Globally, this market is expected to reach 177 billion USD in 2013. The dietary supplements category is expected to be the fastest growing product category globally [2].

## 5. Herbal Medicines in Dietary Supplements

Dietary supplements and herbal remedies are popular complementary or alternative products for people. These are the supplements that are intended to supplement the diet and contain one or more dietary ingredients (including vitamins, minerals, herbs or other botanicals, amino acids, and other substances) or their constituents. These are intended to be taken by mouth as a pill, capsule, tablet, or liquid and are labeled on the front panel as being a dietary supplement. Such products may range from isolated nutrients, dietary supplements, and diets to genetically engineered “designer” foods, herbal products, and processed foods such as cereals, soups, and beverages. These botanicals are sold in many forms as fresh or dried products, liquid or solid extracts, tablets, capsules, powders, tea bags, and so forth. For example, fresh ginger root is often used in various food stores; dried ginger root is sold packaged in tea bags, capsules, or tablets, and liquid preparations made from ginger root are also sold in the market. A particular group of chemicals or a single chemical may be isolated from a botanical and sold as a dietary supplement, usually in tablet or capsule form. An example is phytoestrogens from soy products [36].

## 6. Nutraceutical Concept with Varying Definition

The nomenclature for nutraceuticals is based on the segments it constitutes. In Canada, this term is natural health products; in USA, it is called dietary supplements, and in Japan it is called foods for special health use. There are distinct definitions and regulations for dietary supplements and functional foods in USA, Canada, and Europe. In Japan, dietary supplements and functional foods are governed under the same set of regulations. USA and Canada actually list the constituents that a product must have to be called a nutraceutical, whereas Europe and Japan just provide general guidelines on the properties that a product should have to be called a nutraceutical. Traditional and herbal medicines are included in the definition of dietary or nutritional supplements in Canada. Japan does not mention traditional herbal medicines under functional foods for special health use. USA includes herbal and botanical in its definition. The Indian definition lists down the ingredients that a product should have, and it also specifies general properties of nutraceutical. Traditional medicines though have been excluded from the definition. There are three categories which have been considered under the nutraceuticals [2].

*Functional Foods*. Foods that have specific physiological benefits and/or reduce the risk of chronic disease, that is, nutrition fortified foods like fortified flour, fortified oil, fortified malt-based powder and probiotic foods like yogurt.

*Dietary Supplements*. Supplements provide nutrients that are missing or are not consumed in sufficient quantity in a person's diet, that is, vitamin supplements, mineral supplements, macronutrients, antioxidants, tonics, herbal formulations like Chyawanprash, Musli pak, Ashwagandhadi leh, and nonherbal products like cod liver oil.

*Functional Beverages*. Liquids that quench thirst along with replenishing minerals provide energy, prevent ailments, and promote healthy life style, that is, sports and energy drinks, fortified juices, and glucose drinks and powder. 

A product category can be classified into a specific need-segment based on its predominant use. The product segments catering to foundation and condition specific need are the largest and growing the fastest. Nutraceutical products aim to fulfill specific needs of the persons based on which they may be classified as follows.

*enhancement segments:* high protein supplements, energy drinks, sports drinks, glucose drinks, and so forth. 

*specific condition segments*: antioxidants, vitamin supplements, and mineral supplements.

*foundation segments*: macronutrient supplements, nutrition fortified foods (fortified flour, soups, biscuits, etc.), probiotic foods (yogurt), and herbal formulations (chyawanprash, Ashwagandhadi leh). 

## 7. Conclusion

Although some uncertainty exists about the safety, effectiveness, and cost-effectiveness of CAM methods, expanding their use, where reasonable evidence of their effectiveness and good evidence of their safety exists, might yield health, social, and economic benefits [35]. For example, improving the information and services provided in local pharmacies, that are the primary source of treatment for many ailments in rural areas, might serve as an effective substitute for allowing unregulated use of conventional medical treatment. Thus, expanding CAM would require significant investment of time and resources if it is to be done appropriately and have an impact on population health. An important role exists for CAM. However, more evidence is needed before CAM approaches can be broadly integrated into national health systems for diseases for which they have promise. 

Also, numerous nutraceutical combinations have entered the international market through exploration of ethnopharmacological claims made by different traditional practices. To truly consume a healthy diet, the vast majority of the diet must be composed of health-promoting foods and nutraceuticals but disease-promoting foods or junk food must be avoided. Ninety percent of the daily diet should be made up of nutrient rich plant foods, whose calories are accompanied by health-promoting phytochemicals, vegetables, fresh fruits, beans and legumes, raw nuts, seeds, and avocados, starchy vegetables, and whole grains. These foods or nutraceuticals construct a health-promoting, disease-preventing diet with protective substances. The rich nutrient food intake will provide maximum protection against not only infections, asthma, and allergies but also against heart disease and cancer in adulthood.

## Figures and Tables

**Table 1:** Impact of modern food concept in required nutrition.

Nutrients	Intake by traditional ways	Intake by modern ways	Effect on nutrient intake
Water soluble vitamins (vitamins B and C) and minerals	Vegetables used for cooking were/are fresh	Freezing and packaging of the cut vegetables	Loss of ascorbic acid, water soluble vitamins, and minerals
Proteins, minerals, and vitamin B complex	Manual processing of cereals, without polishing	Milling and polishing of cereals	Reduces protein, minerals, and vitamin B complex
Calcium, iron, thiamine, and niacin	Fresh grinding at home	Heavy milling and poor storage conditions	Loss of calcium, iron, thiamin, and niacin
Iron	Cooking in iron pot	Food generally cooked in cookware like nonstick and Teflon-coated utensils	The benefit of organic iron from the conventional iron pot is not obtained by using modern cookware
Copper	Storing of water and cooking use of copper vessels	Stainless steel utensils and plastic wares	Copper required in minor amount which is not gained from modern utensils used today. Deficiency is known to cause chronic diarrhea, malabsorption problems, and reduce immunity. Use of plastic containers is also harmful

**Table 2:** Some common medicinal plants having nutraceutical potential and their primary use in traditional medicine.

Plant name	Common name	Uses
*Asparagus racemosus *Willd	Shatavari	A potent Ayurvedic rejuvenative. It supplies many female hormones and mostly recommended for those women who have hysterectomies. It also helps to maintain urinary tract and strengthens the immune system and also purifies the blood.

*Commiphora mukul *Engl.	Guggul	A major ingredient in joint and immunocare and regarded as a remedy in Ayurvedic medicine; it increase white blood cell count to possess strong immuno-modulating properties. It also protects against the common cold as well as used in various other conditions like lower cholesterol and triglycerides, while maintaining the HDL to LDL ratio.

*Cyperus scariosus *Br.	Nagarmusta	Useful in supporting healthy genitourinary system and have hepatoprotective properties.

*Garcinia cambogia *Dr	Garcinia	Fruits contain biologically active compounds (−) hydroxycitric acid, which is known to inhibit the synthesis of lipids and fatty acids. HCA inhibits the enzyme ATP-citrate lyase that leads to reduce production of acetyl CoA, which is a key substance in fat and carbohydrate metabolism. Therefore, formation of LDL and triglycerides is very low. It also suppresses appetite by promoting synthesis of glycogen. That way the brain gets signals of fullness and satisfaction sooner. Garcinia contains significant amounts of vitamin C and used as a heart tonic.

*Glycyrrhiza glabra *L.	Yashtimadhu, Licorice	It is a versatile medicine in India and China, for gastrointestinal health. It is a mild laxative, soothes and tones the mucous membranes, and relieves muscle spasms. It is an antioxidant, cancer protecting, botanical boosting, and certain immune functions such as interferon production. Its mode of action is as an antimutagen, preventing damage to genetic material that can eventually result in cancer.

*Gymnema sylvestre *R. Br.	Gurmarar	Its Sanskrit name means literally “sugar destroyer,” has a glycolytic action, and reduces the strength of a glucose solution. It has been used in Ayurveda to regulate sugar metabolism for several centuries. It increases insulin production, regeneration of pancreas cells, and the site of insulin production. Another property is abolishing the taste of sugar, so that Gurmarar has been effective to suppress and neutralize the craving for sweets.

*Melia azadirachta *L.	Nimba, Neem	It has strong health alleviating activity, used as a tonic and astringent that promotes healing. The extract has antispasmodic action. Its usage in Ayurvedic medicine for thousands of years has proved its detoxifying properties. It has shown most beneficial effects for the circulatory, digestive, respiratory, and urinary systems.

*Momordica charantia *L.	Karela, Bitter melon	Karela has been widely used in Ayurvedic medicine. It contains Gurmarin, a polypeptide considered to be similar to bovine insulin, and has a strong sugar regulating effect by suppressing the neural responses to sweet taste stimuli.

*Moringa pterygosperma *Gaertn	Shigru, Horseradish tree	Shigru contains physiologically active principles that is effective in a broad range of health needs. It contains “Pterygospermin,” an antibiotic-like substance.

*Mucuna pruriens *Baker	Kiwanch, Kapikachchhu, Cow-itch plant	It is a good natural source of *L. dopa*. In the Ayurvedic system it is reported as an effective tonic for nervous system. Studies have demonstrated its usefulness maintaining optimum performance of the nervous system.

*Nardostachys jatamansi *DC.	Jatamansi, Musk root	Jatamansi is a relaxing plant, effectiveness for mental health. It is used in various Ayurvedic formulations as a potent ingredient. It has been shown effective in maintaining a restful sleep and with many menopausal symptoms.

*Piper longum *L.	Pippali, Indian Long Pepper	Pippali is a powerful stimulant for both the digestive and the respiratory systems and has a rejuvenating effect on lungs. It plays an important role in release of metabolic heat energy. This effect is the result of increased thyroid hormone level in the body. Pippali a typical Ayurvedic complementary component whose benefit is to increase the bioavailability and enhance absorption of the other active ingredients.

*Piper nigrum *L.	Maricha, Black pepper	The black pepper is one of the most important spices which is widely used to amplify the body's ability to absorb nutrients contained in the food and aid the digestive process.

*Bergenia ligulata *Wall	Pasanavheda	It has the unique property like diuretic action with optimum urinary tract health. This important drug supports bladder by acting on the crystalloid-colloid balance and keeping calcium salts in solution.

*Terminalia chebula *Retz.	Haritaki	Haritaki is a safe and effective purgative, expectorant, and tonic. It is an important ingredient of the classical Ayurvedic formulation “Triphala” which has a combination of three fruits. Tiphalpha is an important Ayurvedic medicine, which promotes health through successive steps of purification and detoxification. It is known to have strong antimutagenic activity, because of its very rich content vitamin C.

*Tinospora cordifolia *Miers	Guduchi	Guduchi is a rich source of natural vitamin C and effective in inhibiting the growth of bacteria and in building up the immune resistance and has immune-boosting ability. Use of this plant increases white blood cells the killing ability of macrophages, the immune cells responsible for fighting invaders.

*Withania somnifera *(L.) Dunal	Ashwagandha	In Ayurvedic medicines Ashwagandha holds a place similar to Ginseng in traditional Chinese medicinal therapies. It is also called the “Indian Ginseng.” It has been used for thousands of years as a popular remedy in Ayurvedic systems for many conditions. It is one of the best health tonics and restorative agents that have been used to treat general debility.

*Zingiber officinale *Rosc	Sunthi, Ginger	Ginger is considered an adjuvant in many Ayurvedic formulas in which it enhances absorption and prevents gastrointestinal side effects. It is a very common spice which is used in Ayurvedic medicine to improve digestion and to prevent nausea. These properties help bowel movements and relax the muscles which control the digestive system.

**Table 3:** Some important herbal formulations frequently used in traditional Ayurvedic system in India.

Disease	Formulation's ingredients/ratio	Dose/method of use
Anemia	*Asparagus racemosus* (roots) 20% *Withania somnifera* (roots) 20% *Phyllanthus emblica* (fruits) 15% *P. amarus* (leaves) 10% *Tephrosia purpurea* (leaves) 10% *Plumbago zeylanica* (roots) 5% *Glycyrrhiza glabra* (roots) 15% *Piper longum* (fruits) 5%	4 gm of powder is given to the patient, twice daily with water

Asthma/bronchitis	*Solanum xanthocarpum* (whole plant) 25% *Piper longum* (fruits) 10% *Adhatoda vasica* (leaves) 25% * Zingiber officinale* (roots) 10% *Curcuma zedoaria* (roots) 10% *Ocimum sanctum* (leaves) 10% *Phyllanthus emblica* (fruits) 10%	4 gm (one teaspoonful) of mixed powder given to the patient, twice a day (morning and at bedtime) with water

Arthritis	*Piper longum* (fruits) 10% *S. xanthocarpum* (whole plant) 15% *Withania somnifera* (roots) 10% *Terminalia chebula* (fruits) 10% *T. bellerica* (fruits) 10% *Curcuma zedoaria* (roots) 15% *Phyllanthus emblica* (fruits) 15% *Ricinus communis* (roots) 15%	4 gm of mixed powder should be given to the patient, twice daily (morning and evening, one hour before meals) with ginger juice for rheumatic problems

Blood circulation	*Zingiber officinale* (roots) 20% *Piper longum* (roots) 10% *Withania somnifera* (roots) 10% *Phyllanthus emblica* (fruits) 10% *Curcuma longa* (roots) 10% *Terminalia bellerica* (fruits) 10% *T. chebula* (fruits) 10% *Ocimum sanctum* (leaves) 10% *Tephrosia purpurea* (leaves) 10%	4 gm of mixed powder is given to the patient, twice daily with water

Cancer	*Azadirachta indica* (bark) 20% *Bauhinia variegata* (bark) 15% *Crataeva nurvala* (bark) 15% *Terminalia chebula* (fruits) 15% *T. bellerica* (fruits) 10% *Holarrhena antidysenterica* (bark) 10% *Tinospora cordifolia* (stems) 15%	4 gm of mixed powder should be given to the patient, twice a day (morning and night) with lukewarm honey for cancer cure

Chronic constipation	*Holarrhena antidysenterica* (bark) 10% *Plumbago ovata* (husk) 20% *Terminalia bellerica* (fruits) 10% *T. chebula* (fruits) 15% *Phyllanthus emblica* (fruits) 15% *Cassia angustifolia* (leaves) 20% *Glycyrrhiza glabra* (roots) 10%	4 gm of mixed powder is given to the patient, at night before going to bed, with water

Chronic fever	*Tinospora cordifolia* (stems) 15% *Ocimum sanctum* (leaves) 15% *Adhatoda vasica* (leaves) 15% *Azadirachta indica* (leaves) 15% *Holarrhena antidysenterica* (bark) 10% *Piper longum* (fruits) 10% *Zingiber officinale* (roots) 10% *Terminalia bellerica* (fruits) 10%	4 gm of mixed powder is given to the patient, twice daily before meals with water.

Cough	*Phyllanthus emblica* (fruits) 25% *Adhatoda vasica* (leaves) 20% *Ocimum sanctum* (leaves) 10% *Piper longum* (fruits) 10% *Zingiber officinale* (roots) 10% *Glycyrrhiza glabra* (roots) 15% *Solanum xanthocarpum* (whole plant) 10%	3 gm of mixed powder should be given to the patient twice daily (morning and at night before going to bed) with lukewarm mixed with honey to cure cold

Cysts	*Terminalia chebula* (fruits) 20% *Azadirachta indica* (bark) 20% *Holarrhena antidysenterica* (bark) 10%, *Terminalia bellerica* (fruits) 10% *Withania somnifera* (roots) 20% *Tinospora cordifolia* (stems) 20%	4 gm of mixed (one teaspoonful) powder is given to the patient, twice a day (morning and evening) with water

Dental diseases	*Azadirachta indica* (leaves) 15% *A. arabia* (bark) 15% *Areca catechu* (bark) 15% *Achyranthes aspera* (leaves) 10% *Ficus benghalensis* (bark) 15% *Quercus infectoria* (fruits) 15% *Symplocos racemosa* (bark) 15%	The powder is applied to the gums and teeth, two times a day. Additionally a gargle of the decoction (3 gm of powder mixed in 150 mL of water)

Diarrhoea	*Holarrhena antidysenterica* (bark) 25% *Aegle marmelos* (fruits) 25% *Zingiber officinale* (roots) 10% *Terminalia chebula* (fruits) 10% *Cyperus rotundus* (roots) 10% *Syzygium cumini* (seeds) 10% *Phyllanthus emblica* (fruits) 10%	3 gm of mixed powder is given to the patient, three times a day, with curd for dysentery and diarrhoea

Dislocation of bones	*Asparagus racemosus* (roots) 15% *Withania somnifera* (roots) 15% *Azadirachta arabica* (bark) 20% *Terminalia arjuna* (bark) 20% *T. chebula* (fruits) 10% *T. bellerica* (fruits) 10% *Phyllanthus emblica* (fruits) 10%	3 gm of mixed powder is given to the patient, twice a day with water for dislocation of bones and fractures

Diabetes	*Gymnema sylvestre* (leaves) 30% *Tinospora cordifolia* (stems) 15% *Azadirachta indica* (leaves) 10% *Phyllanthus emblica* (fruits) 20% *Curcuma longa* (roots) 10% *Aegle marmelos* (leaves) 15%	4 gm of mixed powder should be given to the patient, twice a day with water

Fistula	*Glycyrrhiza glabra *(roots) 20% *Terminalia chebula* (fruits) 20% *T. bellerica* (fruits) 15% *Tinospora cordifolia* (stems) 15% *Azadirachta indica* (leaves) 15% *Withania somnifera* (roots) 15%	3 gm of mixed powder should be given to the patient, twice daily with water to treat fistula

Female sterility	*Asparagus racemosus* (roots) 20% *Withania somnifera* (roots) 20% *Glycyrrhiza glabra* (roots) 20% *Phyllanthus emblica* (fruits) 10% *Ficus glomerata* (bark) 10% *F. religiosa* (bark) 10%	3 gm of mixed powder is given to the patient twice daily, half an hour before meals with milk

General health tonic	*Withania somnifera* (roots) 20% *Asparagus racemosus* (roots) 10% *Glycyrrhiza glabra* (roots) 10% *Tribulus terrestris* (fruits) 10% *Phyllanthus emblica* (fruits) 15% *Terminalia arjuna* (bark) 15% *Centella asiatica* (leaves) 10%	4 gm of powder is given to the patient, twice daily (morning and evening) with milk

Gastritis	*Zingiber officinale* (roots) 10% *Piper longum* (fruits) 10% *Mentha piperita* (leaves) 10% *Terminalia chebula* (fruits) 15% *T. bellerica* (fruits) 15% *Phyllanthus emblica* (fruits) 15% *Plumbago zeylanica* (roots) 10% *Tinospora cordifolia* (stems) 15%	4 gm of (one teaspoonful) mixed powder is given to the patient twice daily, half an hour before meals with water

Hair problems	*Eclipta alba* (leaves) 15% *Centella asiatica* (leaves) 15% *Terminalia chebula* (fruits) 10% *T. bellerica* (fruits) 10% *Phyllanthus emblica* (fruits) 15% *Glycyrrhiza glabra* (roots) 15% *Tinospora cordifolia* (stems) 10% *Tribulus terrestris* (fruits) 10%	4 gm of mixed powder is given to the patient, twice a daily with honey

High blood pressure	*Terminalia arjuna* (bark) 35% *T. chebula* (fruits) 15% *Asparagus racemosus* (roots) 15% *Zingiber officinale* (roots) 10% *Withania somnifera* (roots) 25%	4 gm of powder is given to the patient, twice a day (morning and night) with honey

Heart tonic	*Withania somnifera* (roots) 10% *Terminalia arjuna* (bark) 30% *T. bellerica* (fruits) 10% *T. chebula* (fruits) 10% *Cyperus rotundus* (roots) 10% *Phyllanthus emblica* (fruits) 10% *Ocimum sanctum* (leaves) 10%	3 gm of mixed powder is given to the patient, twice a day with water

Intestinal worms	*Holarrhena antidysenterica* (bark) 10% *Mentha piperita* (leaves) 10% *Tinospora cordifolia* (stems) 20% *Butea monosperma* (seeds) 20% *Azadirachta indica* (leaves) 10% *Phyllanthus emblica* (fruits) 20% *Tribulus terrestris* (fruits) 10%	3 gm of mixed powder is given to the patient, twice daily (morning and night) with water

Epilepsy	*Centella asiatica* (leaves) 30% *Withania somnifera* (roots) 20% *Tribulus terrestris* (fruits) 15% *Piper longum* (roots) 10% *Achyranthes aspera* (leaves) 15% *Plumbago zeylanica* (roots) 10%	3 gm mixed powder is given to the patient, twice daily (morning and evening) with fruit juice to treat Hysteria

Leucorrhoea	*Symplocos racemosa* (bark) 35% *Asparagus racemosus* (roots) 15% *Adhatoda vasica* (leaves) 10% *Aegle marmelos* (fruits) 10% *Phyllanthus emblica* (fruits) 10% *Azadirachta indica* (bark) 10%	3 gm of mixed powder is given to the patient, twice daily with water

Leucoderma	*Psoralea corylifolia* (seeds) 20% *Terminalia chebula* (fruits) 10% *Phyllanthus emblica* (fruits) 20% *Azadirachta indica* (bark) 20% *Areca catechu* (bark) 10% *Tinospora cordifolia* (stems) 10% *Eclipta alba* (leaves) 10%	3 gm of mixed powder should be given to the patient, twice a day before meals with water

Liver tonic	*Holarrhena antidysenterica* (bark) 10% *Eclipta alba* (leaves) 20% *Tephrosia purpurea* (leaves) 20% *Tinospora cordifolia* (stems) 10% *Azadirachta indica* (bark) 10% *Phyllanthus amarus* (whole plant) 20% *Plumbago zeylanica* (roots) 10%	4 gm of mixed powder is given to the patient twice daily, half an hour before meals with water

Lack of appetite	*Zingiber officinale* (roots) 10% *Piper longum* (fruits) 10%, *Phyllanthus emblica* (fruits) 30% *Terminalia chebula* (fruits) 15% *Tinospora cordifolia* (stems) 15% *Cassia angustifolia* (leaves) 10% *Mentha piperita* (leaves) 10%	4 gm of mixed powder is given to the patient, two times a day after meals with water for indigestion

Male sterility	*Withania somnifera* (roots) 15% *Mucuna pruriens* (seeds) 25% *Tribulus terrestris* (fruits) 20% *Glycyrrhiza glabra* (roots) 10% *Terminalia arjuna* (bark) 10% *Phyllanthus emblica* (fruits) 10% *Zingiber officinale* (roots) 5% *Piper longum* (fruits) 5%	4 gm of mixed powder is given to the patient, twice a day with honey

Migraine	*Curcuma longa* (roots) 15% *Glycyrrhiza glabra* (roots) 15% *Azadirachta indica* (bark) 15% *Tinospora cordifolia* (stems) 15% *Terminalia chebula* (fruits) 10% *Ocimum sanctum* (leaves) 15% *Eclipta alba* (leaves) 15%	4 gm of mixed powder is given to the patient, twice a day with honey

Obesity	*Terminalia chebula* (fruits) 15% *Terminalia bellerica* (fruits) 15% *Phyllanthus emblica* (fruits) 10% *Crataeva nurvala* (bark) 25% *Tribulus terrestris* (fruits) 25% *Zingiber officinale* (roots) 10%	4 gm of powder is given to the patient, twice a day with warm water

Paralysis	*Curcuma zedoaria* (roots) 20% *Withania somnifera* (roots) 20% *Tribulus terrestris* (fruits) 20% *Zingiber officinale* (roots) 20% *Piper longum* (fruits) 5% *Crataeva nurvala* (leaves) 10% *Plumbago zeylanica* (roots) 5%	3 gm of mixed powder is given to the patient, three times a day with honey

Prostate enlargement	*Tinospora cordifolia* (stems) 15% *Tribulus terrestris* (fruits) 15% *Phyllanthus emblica* (fruits) 15% *Zingiber officinale* (roots) 10% *Butea monosperma* (seeds) 10% *Adhatoda vasica* (leaves) 5% *Terminalia chebula* (fruits) 10% *T. bellerica* (fruits) 10% *Glycyrrhiza glabra* (roots) 10%	4 gm of mixed powder is given to the patient twice a day, morning and evening before meals with water

Piles	*Eclipta alba* (leaves) 35% *Terminalia chebula* (fruits) 15% *Terminalia bellerica* (fruits) 10% *Phyllanthus emblica* (fruits) 10% *Adhatoda vasica* (leaves) 10% *Plumbago zeylanica* (roots) 5% *Piper longum* (fruits) 5% *Aegle marmelos* (fruits) 10%	4 gm of mixed powder is given to the patient, twice daily (morning and at bedtime) with water

Sleeplessness	*Withania somnifera* (roots) 20% *Centella asiatica* (leaves) 30% *Piper longum* (roots) 20% *Glycyrrhiza glabra* (roots) 10% *Terminalia bellerica* (fruits) 10%	3 gm mixed powder is given to the patient, at night before going to bed, with milk

Skin diseases	*Cyperus rotundus *(roots) 10% *Tinospora cordifolia* (stems) 20% *Azadirachta indica* (bark) 20% *Terminalia chebula* (fruits) 10% *T. bellerica* (fruits) 10% *Curcuma longa* (roots) 10% *Phyllanthus emblica* (fruits) 10% *Centella asiatica* (leaves) 10%	3 gm of powder is given to the patient, twice a day before meals with water to cure allergy problems

Sexual debility	*Withania somnifera* (roots) 10% *Mucuna pruriens* (seeds) 20% *Asparagus racemosus* (roots) 10% *Sida cordifolia* (seeds) 10% *Tribulus terrestris* (fruits) 20% *Glycyrrhiza glabra* (roots) 10%	About 4 gm of mixed powder should be given to the patient, twice daily (morning and at night before going to bed) with milk

Throat diseases	*Glycyrrhiza glabra* (roots) 30% *Terminalia chebula* (fruits) 10% *T. bellerica* (fruits) 10% *Solanum xanthocarpum* (whole plant) 20% *Piper longum* (fruits) 10% *Sida cordifolia* (roots) 10% *Phyllanthus emblica* (fruits) 10%	4 gm of mixed powder is given to the patient twice daily, morning and at bedtime with honey

Thyroid problems	*Crataeva nurvala* (bark) 20% *Bauhinia variegata* (bark) 20% *Sida cordifolia* (leaves) 15% *Terminalia chebula* (fruits) 10% *T. bellerica* (fruits) 10% *Glycyrrhiza glabra* (roots) 15% *Zingiber officinale* (roots) 10%	3 gm of mixed powder is given to the patient, twice daily with lukewarm water

Urinary tract	*Tribulus terrestris* (fruits) 25% *Zingiber officinale* (roots) 10% *Solanum xanthocarpum* (whole plant) 10% *Crataeva nurvala* (bark) 25% *Tinospora cordifolia* (stems) 10% *Asparagus racemosus* (roots) 10% *Tephrosia purpurea* (leaves) 10%	4 gm of mixed powder is given to the patient, twice a day with water
